# Genome-Wide Identification of *NLP* Gene Families and Haplotype Analysis of *SiNLP2* in Foxtail Millet (*Setaria italica*)

**DOI:** 10.3390/ijms252312938

**Published:** 2024-12-02

**Authors:** Yanming Bai, Juncheng Wang, Wensi Tang, Daizhen Sun, Shuguang Wang, Kai Chen, Yongbin Zhou, Chunxiao Wang, Jun Chen, Zhaoshi Xu, Ming Chen, Huajun Wang, Youzhi Ma

**Affiliations:** 1 College of Agronomy, Gansu Agricultural University, Lanzhou 730070, China; bym_2021@163.com (Y.B.); bcli@gsau.edu.cn (J.W.); 2 State Key Laboratory of Aridland Crop Science, Gansu Key Laboratory of Crop Improvement & Germplasm Enhancement, Gansu Agricultural University, Lanzhou 730070, China; 3 State Key Laboratory of Crop Gene Resources and Breeding, Institute of Crop Sciences, Chinese Academy of Agricultural Sciences (CAAS), Beijing 100081, China; tang_wensi@yeah.net (W.T.); chenkai8@vip.163.com (K.C.); zhouyongbin@caas.cn (Y.Z.); wangchunxiao@caas.cn (C.W.); chenjun01@caas.cn (J.C.); xuzhaoshi@caas.cn (Z.X.); chenming02@caas.cn (M.C.); 4 Key Laboratory of Sustainable Dryland Agriculture, College of Agriculture, Shanxi Agricultural University, Jinzhong 030801, China; sdz64@126.com (D.S.); wsg6162@126.com (S.W.)

**Keywords:** millet, nitrogen, *NLP* transcription factor, haplotype

## Abstract

Nitrogen is a critical factor in plant growth, development, and crop yield. NODULE-INCEPTION-like proteins (NLPs), which are plant-specific transcription factors, function as nitrate sensors and play a vital role in the nitrogen response of plants. However, the genome-wide identification of the *NLP* gene family, the elucidation of the underlying molecular mechanism governing nitrogen response, and haplotype mining remain elusive in millet. In this study, we identified seven members of the *NLP* gene family in the millet genome and systematically analyzed their physicochemical properties. Evolutionary tree analysis indicated that *SiNLP* members can be classified into three subgroups, with *NLP* members from the same species preferentially grouped together within each subgroup. Analysis of gene structure characteristics revealed that all *SiNLP* members contained 10 conserved motifs, as well as the RWP-RK and PB1 domains, indicating that these motifs and domains have been relatively conserved throughout evolution. Additionally, we identified a significant abundance of response elements related to hormones, stress, growth, and development within the promoter regions of *SiNLP* members, suggesting that these members are involved in regulating diverse physiological processes in millet. Transcriptome data under low-nitrogen conditions showed significant differences in the expression profiles of *SiNLP2* and *SiNLP4* compared to the other members. RNA-seq and qRT-PCR results demonstrated that *SiNLP2* significantly responds to low-nitrogen stress. Notably, we found that *SiNLP2* is involved in nitrogen pathways by regulating the expression of the *SiNAR2.1A*, *SiNAR2.1B*, *SiNRT1.1*, and *SiNR2* genes. More importantly, we identified an elite haplotype, Hap2, of *SiNLP2*, which is gradually being utilized in the breeding process. Our study established a foundation for a comprehensive understanding of the *SiNLP* gene family and provided gene resources for variety improvement and marker-assisted selection breeding.

## 1. Introduction

As one of the essential macronutrients for plants, nitrogen is crucial for the growth and development of crops and plays a vital role in determining yield [1,2,3,4]. In soil, nitrogen primarily exists as nitrate and ammonium. Over the course of long-term evolution, plants have developed various nitrate transport systems to adapt to the complex external ecological environment and fluctuations in nitrogen availability. These systems include both low-affinity and high-affinity nitrate transport mechanisms [5]. The *NRT1* family is generally known as a low-affinity nitrate transport system; however, studies have demonstrated that *NRT1.1* functions as a dual-affinity nitrate transporter, with the phosphorylation of threonine (101) being key to its affinity conversion [6]. In contrast, the *NRT2* family consists of high-affinity nitrate transporters [7,8], with *NRT2.1* playing an essential role in the uptake of nitrate by roots [9,10]. Numerous genes are involved in nitrogen absorption, transport, assimilation, and reuse. For example, *ARN* was the first nitrate-responsive transcription factor discovered in Arabidopsis [11]. Subsequently, many key transcription factors associated with nitrate response have been identified, including *NLP6*, *SPL9*, *TGA1*, *NAC4*, *HRS1*, *TCP20*, *LBD37/38/39*, and *NRG2* [12,13,14,15,16,17]. Among these, *NLP7* is one of the most critical transcription factors in the nitrogen pathway, facilitating nitrate response through a nuclear retention mechanism [18,19]. *NLP7* is essential for nitrate signaling and acts as a cellular nitrate sensor [20].

The *NLP* gene family consists of plant-specific transcription factors that play an essential role in plant growth and development, stress response, and nitrogen regulation [21]. This gene family features two typical domains: RWP-RK and PB1. The RWP-RK domain recognizes nitrate-responsive cis-elements (NREs), thereby mediating DNA binding and regulating gene expression in middle and downstream nitrogen pathways [22,23,24]. The PB1 domain is crucial for mediating protein interactions and forming dimers between proteins [22,25]. Additionally, there is generally a GAF domain present at the nitrogen end of *NLP*, which plays an important role in nitrate-dependent transcriptional activity [24]. The *NLP* family has been studied in various species, including *Arabidopsis*, rice, tomato, wheat, *Brassica napus*, Asian cotton, Raymond’s cotton, upland cotton, island cotton, poplar, and bamboo [26,27,28,29,30,31,32]. Research has shown that after nitrate signals are conducted through *NRT1.1*, the activity of phospholipase C (PLC) is activated, resulting in changes in calcium ion concentration. Calcium-dependent protein kinases (CPKs) then decode these calcium signal changes to phosphorylate NLP7 and activate the expression of downstream target genes in the nitrate pathway [33,34]. *AtNLP7* affects lateral root development by regulating the expression of *BT1* and *BT2* [35]. Furthermore, overexpression of *AtNLP7* can promote the growth of both primary and lateral roots [36]. Under nitrate conditions, *AtNLP8* activates the expression of genes in the middle and downstream nitrogen pathways, regulates abscisic acid (ABA) accumulation, and promotes seed germination [37,38]. Under low-nitrogen conditions, zmnlp5 plays a critical role in regulating root growth in maize, and a mutation in zmnlp5 can inhibit root growth [39]. *OsNLP1* binds to the promoter regions of the essential genes *OsNRT1.1A*, *OsNRT1.1B*, and *OsGRF4* in the nitrogen pathway, regulating their expression and participating in nitrogen response. Overexpression of *OsNLP1* can enhance crop yield and nitrogen use efficiency (NUE) [40,41]. These findings indicate that the *NLP* family plays a significant role in plant growth and development, environmental adaptation, and nitrogen response across different species.

Millet (*Setaria italica*) is an important food crop worldwide. As a diploid species, millet has a small genome that is relatively easy to study and exhibits beneficial traits such as drought resistance, tolerance to poor soils, and low requirements for water and fertilizer [42,43,44]. The *NLP* gene family plays a crucial role in plant growth, development, and nitrogen pathways. However, there are few reports on the *NLP* genes in millet. This study identified members of the *NLP* gene family in the millet genome and systematically analyzed their physicochemical properties, evolutionary relationships, conserved gene motifs, functional domains, gene structures, promoter cis-acting elements, and replication events. We also investigated the expression profiles of the *SiNLP* gene family under low-nitrogen stress. Using quantitative reverse transcription PCR (qRT-PCR), we examined the expression of differentially expressed essential genes in the root system at various time points under both normal and low-nitrogen treatment conditions. The key candidate gene *SiNLP2* is involved in the nitrogen pathway by regulating the expression of the *SiNAR2.1A*, *SiNAR2.1B*, *SiNRT1.1*, and *SiNR2* genes. Additionally, we identified the superior haplotype Hap2 and found that it was selected during domestication and improvement. Our study lays the foundation for a deeper understanding of how *SiNLP* responds to nitrogen and provides valuable gene resources and theoretical support for variety improvement and marker-assisted selection in breeding efforts.

## 2. Results

### 2.1. Identification of SiNLP Gene Family Members

Using nine NLP sequences from *Arabidopsis* as query sequences, we identified 42 members of the *SiNLP* gene family through BLAST comparison and HMMER analysis after removing redundancy. We then further verified these results using SMART-v9.0 website, retaining only those members that contained both the RWP-RK (PF02042) and PB1 (PF00564) domains, ultimately identifying seven *SiNLP* gene family members. These members are named according to their physical location on the genome chromosomes [45,46] (Table 1).

### 2.2. Characteristics and Chromosome Localization of SiNLP Gene Family Members

**Figure 1:**
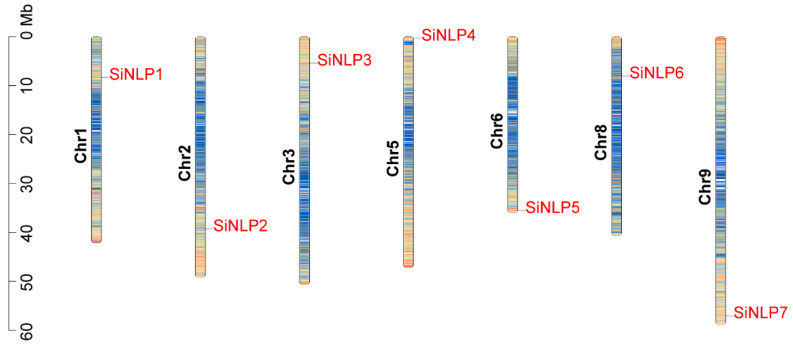
Chromosome localization of *SiNLP* gene family members in millet. Left scale: megabases (Mb).

The amino acid lengths of the *SiNLP* gene family members range from 749 aa (*SiNLP1*) to 1069 aa (*SiNLP5*). The molecular weights vary from 79,534.4 Da (*SiNLP1*) to 117,460.25 Da (*SiNLP5*), and the theoretical isoelectric point (pI) ranges from 5.38 to 6.29, indicating that all proteins are acidic. Additionally, the instability index for these proteins ranges from 42.28 to 51.49, with all values exceeding 40. The aliphatic index varies between 73.97 and 84.36, with an average value of 77.69. The grand average of hydropathicity ranges from −0.433 to −0.262, both of which are less than 0, suggesting that all *SiNLP* gene family proteins are hydrophilic. Subcellular localization prediction results indicate that, except for the protein encoded by the *SiNLP1* gene, which is located in the chloroplast, the proteins encoded by the other genes are all located in the nucleus (Table 1). To understand the distribution of *SiNLP* gene family members on chromosomes, we mapped them onto millet chromosomes. The results showed that seven *SiNLP* gene family members are distributed across chr1, chr2, chr3, chr5, chr6, chr8, and chr9 (Figure 1).

### 2.3. Phylogenetic and Sequence Analyses of the SiNLP Gene Family

**Figure 2:**
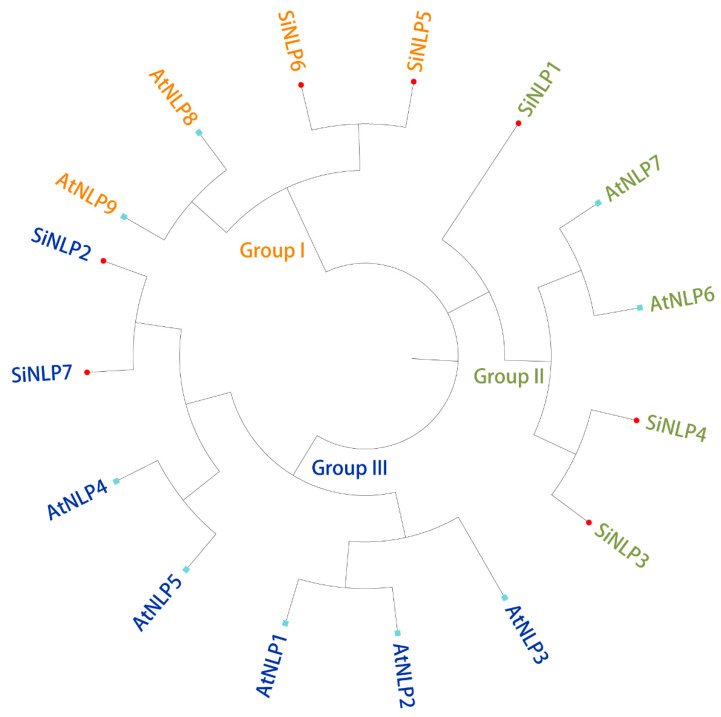
NLP evolutionary tree analysis of *Arabidopsis* and millet. Different colors represent different subgroups. The red dots indicate the *NLP* gene in millet, while the light blue dots represent the *NLP* gene in *Arabidopsis*.

To understand the evolution of the *SiNLP* gene family in millet, we used nine *NLP* family members from *Arabidopsis* as a reference and employed MEGA-X-10.1.8 software to perform multiple-sequence comparisons between *SiNLP* gene family members and *Arabidopsis NLP* family members. We constructed the evolutionary tree using the Neighbor-Joining method. The results indicated that the tree could be divided into three subgroups. The first subgroup contained two *Arabidopsis* NLPs (*AT3G59588.1* and *AT2G43500.2*) and two SiNLPs (*SiNLP5* and *SiNLP6*). The second subgroup included two *Arabidopsis* NLPs (*AT4G24020.1* and *AT1G64530.1*) and three SiNLPs (*SiNLP1*, *SiNLP3*, and *SiNLP4*). The third subgroup consisted of five *Arabidopsis* NLPs (*AT1G20640.1*, *AT1G76350.1*, *AT2G17151.1*, *AT4G35270.1*, and *AT4G38340.1*) and two SiNLPs (*SiNLP2* and *SiNLP7*). Within each subgroup, *NLP* members from the same species are preferentially clustered together (Figure 2).

### 2.4. Analysis of Conserved Motifs, Domains, Gene Structure, and Cis-Acting Elements of the SiNLP Gene Family

**Figure 3:**
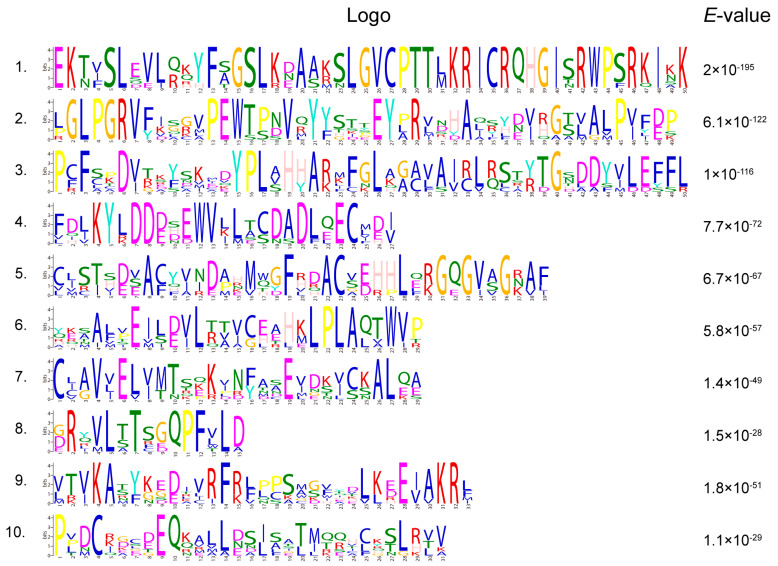
Logos of the ten conserved motifs of *SiNLP* gene family members.

**Figure 4:**
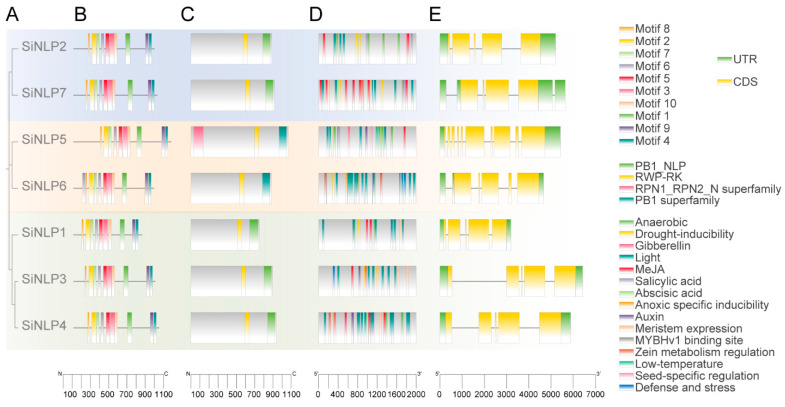
Analysis of the phylogenetic tree, conserved motifs, domains, cis-acting elements, and gene structure of the millet *SiNLP* family. (**A**) Phylogenetic tree analysis of the seven *SiNLP* family members. (**B**) Analysis of conserved motifs. (**C**) Domain analysis. (**D**) Distribution of cis-acting elements in the promoter regions of the *SiNLP* family. (**E**) Gene structure analysis.

To understand the structural characteristics, function, and evolutionary relationships of the *SiNLP* gene family, we conducted various analyses. The conserved motif analysis identified motifs 1–10, corresponding to logos 1–10 (Figure 3). The results indicated that all members of the *SiNLP* gene family contain all ten motifs, suggesting that these motifs are highly conserved throughout evolution (Figure 4A,B). The domain analysis of SiNLPs showed that all seven *SiNLP* family members contain RWP-RK and PB1 domains, while all other *NLP* members, except for *SiNLP7*, also contain GAF domains (Figure 4C, Table S1). To gain a comprehensive understanding of the gene structure within the *SiNLP* gene family, we examined the arrangement and distribution of UTRs, exons, and introns for all members. The results revealed that the *SiNLP7* gene contains two 5′ UTR and two 3′ UTR, while the *SiNLP6* gene has two 5′ UTR and one 3′ UTR. All other genes have one 5′ UTR and one 3′ UTR. Among them, *SiNLP5* has the highest number of exons (10), *SiNLP7* has the lowest (4), and the other genes have 5 exons each. These findings suggest that functional differentiation occurred among the *SiNLP* gene family members during evolution to some extent (Figure 4E).

To determine the pathways in which the *SiNLP* gene family is involved, we analyzed the cis-acting elements located within the 2000 bp promoter region upstream of the *SiNLP* genes. The results revealed that the *SiNLP* gene family primarily contains hormone response elements, including auxin response elements such as TGA-element and AuxRR-core, as well as erythromycin response elements like P-box, ABRE, and TCA-element. Additionally, the analysis identified stress response elements, including TC-rich repeats associated with defense and stress responses, LTR for low-temperature response, ARE for anaerobic induction, and MBS for drought response. Furthermore, growth and development response elements such as CAT-box, along with light response elements like AE-box, I-box, GATA-motif, and G-box, were also present. These findings indicate that the expression of *SiNLP* gene family members is induced by hormones, stress, growth and developmental cues, and light signals, thus suggesting their participation in related pathways (Figure 4D).

### 2.5. Collinearity Analysis of the NLP Gene Family

**Figure 5:**
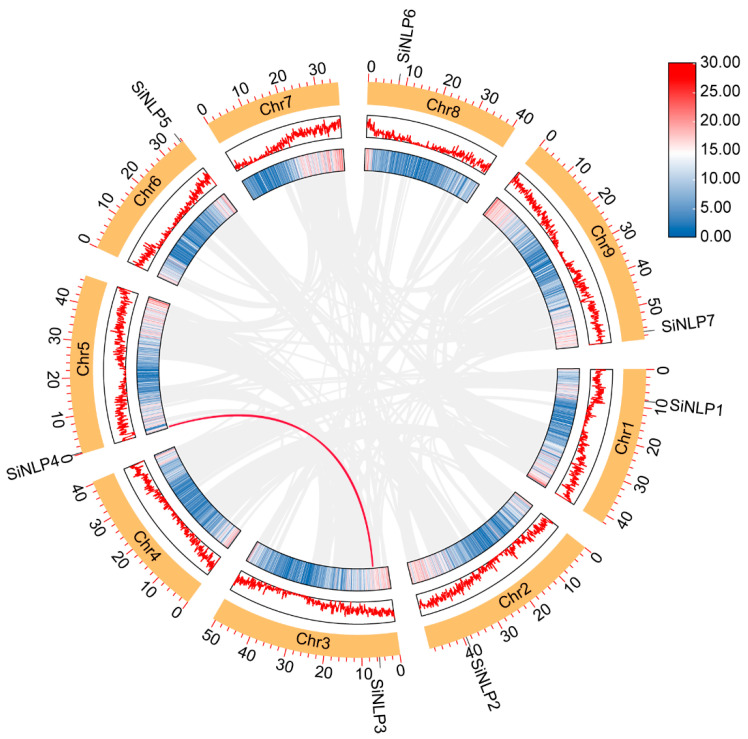
Collinearity analysis of millet *SiNLPs*. The red lines represent collinear gene pairs, while the numbers in the orange boxes indicate chromosomes. Both the red lines and the gradient shading within the boxes represent gene density.

**Figure 6:**
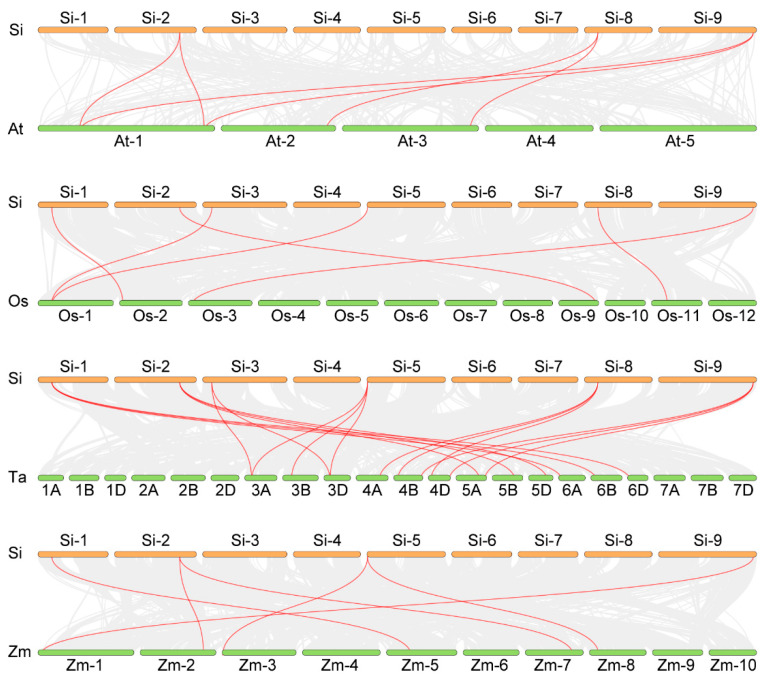
Collinearity analysis of *NLPs* between millet and other species. The red lines represent interspecies collinear gene pairs, with “Si” for millet, “At” for *Arabidopsis*, “Os” for rice, “Ta” for wheat, and “Zm” for maize.

In the process of crop evolution, genome replication events play a crucial role in the expansion of gene families and the formation of new ones, thereby affecting the adaptability and growth of crops. We used MCScanX to analyze the concatenation and fragment replication within the millet genome. Our analysis revealed that the *SiNLP* gene family contains one collinear gene pair, *SiNLP3* and *SiNLP4*, located on chromosomes three and five, respectively (Figure 5). To further understand the homology and evolutionary mechanisms of the *NLP* gene family in millet and other species, we analyzed the collinearity of the *NLP* gene family in millet with that of *Arabidopsis*, rice, wheat, and maize. The results showed seventeen collinear gene pairs between millet and wheat, as well as six collinear gene pairs between millet and *Arabidopsis*, rice, and maize (Figure 6).

### 2.6. Expression Profile Analysis of the SiNLP Gene Family and qPCR of Candidate Genes

**Figure 7:**
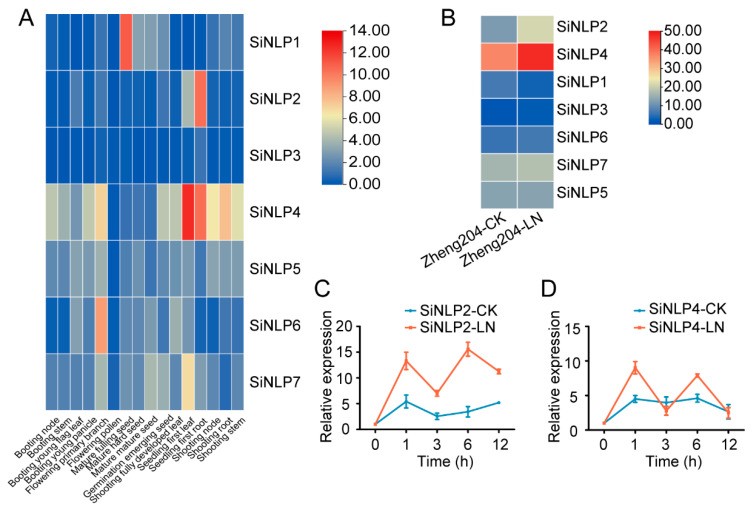
Transcriptome expression profile of millet *SiNLPs* and qPCR analysis of candidate genes. (**A**) Transcriptome expression profiles of *SiNLPs* in different tissues of foxtail millet; (**B**) transcriptome expression profiles of *SiNLP* gene family members under low-nitrogen conditions in millet; (**C**) expression levels of *SiNLP2* in roots at different time points under normal and low-nitrogen treatment conditions; and (**D**) expression levels of *SiNLP4* in roots at different time points under normal and low-nitrogen treatment conditions. CK: normal treatment (1 mM Ca(NO3)_2_), LN: low-nitrogen treatment (0.1 mM Ca(NO3)_2_).

Firstly, we analyzed the expression profiles of the *SiNLP* gene family in different tissues. The results indicated that *SiNLP2* and *SiNLP4* were mainly expressed in roots, *SiNLP1* was mainly expressed in seeds, *SiNLP6* was predominantly expressed in branches, and *SiNLP7* was primarily expressed in leaves (Figure 7A). Next, we examined the expression of *SiNLP* gene family members using transcriptome data from millet variety Zheng204 under low-nitrogen stress, published by our laboratory. The analysis revealed that the following five genes in the *SiNLP* family showed up-regulated expression: *SiNLP2*, *SiNLP3*, *SiNLP4*, *SiNLP6*, and *SiNLP7*, while *SiNLP1* and *SiNLP5* were down-regulated (Figure 7B). Notably, the expression of *SiNLP2* was considerably up-regulated following low-nitrogen treatment, with a *p*-value of 1.37 × 10^−36^ (Table S2). To further investigate the expression of *SiNLP* gene family members in roots at different time points under normal and low-nitrogen stress, we collected root samples at 0, 1, 3, 6, and 12 h post-treatment. We performed qPCR analysis on the two candidate genes with the highest up-regulated expression. The results showed that the expression of *SiNLP2* was significantly induced by low nitrogen, peaking at 6 h (Figure 7C). In contrast, the expression of *SiNLP4* slightly decreased at 3 h compared to normal conditions, but was generally up-regulated under low nitrogen at the other time points (Figure 7D). These findings are consistent with the transcriptome results.

### 2.7. The DLR Analysis Between SiNLP2 and Nitrogen Pathway Genes

**Figure 8:**
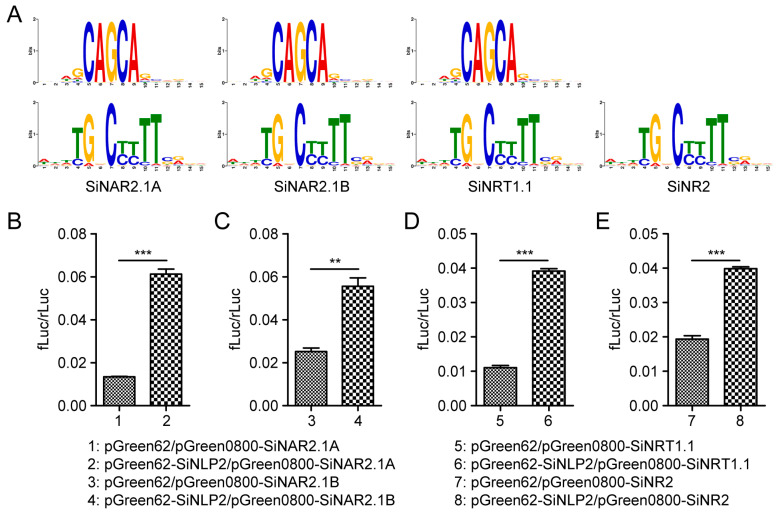
Prediction of cis-acting elements in the promoter regions of key genes in the nitrogen pathway and DLR assay. (**A**) *NLP* transcription factor binding assay for the promoter regions of *SiNAR2.1A*, *SiNAR2.1B*, *SiNRT1.1*, and *SiNR2*. (**B**) DLR assay for *SiNLP2* and *SiNAR2.1A*. (**C**) DLR assay for *SiNLP2* and *SiNAR2.1B*. (**D**) DLR assay for *SiNLP2* and *SiNRT1.1*. (**E**) DLR assay for *SiNLP2* and *SiNR2*. Results were analyzed through students’ *t*-test for statistical significance. In this result, ** represents *p*-value ≤ 0.01, and *** *p*-value ≤ 0.001.

The expression of the *SiNLP2* gene was significantly up-regulated following low-nitrogen induction, indicating that it plays a role in nitrogen pathways. To further investigate the function of candidate genes in these pathways, we extracted key nitrogen pathway genes identified in previous studies. The results revealed that the promoter regions of the *SiNAR2.1A*, *SiNAR2.1B*, *SiNRT1.1*, and *SiNR2* genes all contained binding motifs for *NLP* transcription factors, with the conserved motifs being TGCTG and CTTTT (Figure 8A). Additionally, through double luciferase reporter (DLR) assay experiments, we found that the *SiNLP2* gene could bind to the promoters of *SiNAR2.1A*, *SiNAR2.1B*, *SiNRT1.1*, and *SiNR2*, promoting their transcription (Figure 8B–E).

### 2.8. Haplotype Analysis of the Candidate Gene SiNLP2

**Figure 9:**
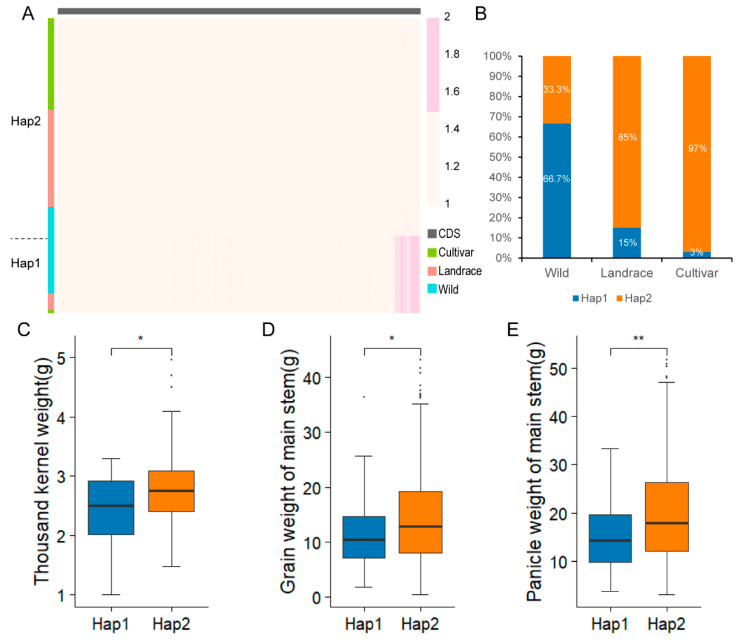
Haplotype analysis of the candidate gene *SiNLP2*. (**A**) Heat maps illustrating the multiple-sequence alignment of *SiNLP2* across 103 millet germplasm samples. Light yellow indicates reference (Ref), while light red indicates alternative (Alt). Hap1 represents haplotype 1, Hap2 represents haplotype 2, Wild indicates wild varieties, Landrace indicates landrace varieties, and Cultivar indicates modern cultivar varieties. (**B**) Distribution of different haplotypes during domestication and improvement. (**C**–**E**) Analysis of significant differences in thousand-kernel weight, grain weight of the main stem, and panicle weight of the main stem among different haplotypes. Results were analyzed through students’ *t*-test for statistical significance. In this result, * represents *p*-value ≤ 0.05, and ** *p*-value ≤ 0.01.

To further understand the evolutionary mechanism of *SiNLP2*, an essential candidate gene of the *SiNLP* gene family, we analyzed 103 core germplasm materials with rich genetic diversity, including 30 wild varieties, 40 landrace varieties, and 33 cultivar varieties. We identified several single nucleotide polymorphisms (SNPs), insertions and deletions (Indels), and presence/absence variations (PAVs) in the coding sequence (CDS) region. The results indicated that these markers were linked to some extent, allowing us to classify them into two main haplotypes (Figure 9A). Interestingly, we found that haplotype Hap2 appeared to have been selected with an increasing trend during domestication and improvement, transitioning from wild varieties to landrace varieties and then to cultivar varieties. The frequency of Hap2 increased dramatically during domestication, from 33.3% to 85%, and at a slower rate during improvement, from 85% to 97% (Figure 9B). We further analyzed yield traits associated with the two different haplotypes, including thousand kernel weight, grain weight of the main stem, and panicle weight of the main stem. The results showed that the thousand kernel weight, grain weight of the main stem, and panicle weight of the main stem of Hap2 were significantly greater compared to those of Hap1, indicating that *SiNLP2* is not only related to nitrogen response but also to yield traits. The superior Hap2 haplotype has been widely utilized in modern breeding programs (Figure 9C–E).

## 3. Discussion

Nitrogen, as one of the essential macronutrients, plays a crucial role in plant growth, development, and yield improvement [47]. However, excessive nitrogen application can lead to environmental issues, such as soil degradation and water pollution [48]. As an important food crop, millet is widely recognized for its beneficial traits, including tolerance of poor soil conditions and low water and fertilizer availability [44]. Consequently, using millet to explore nitrogen-utilization-related genes is an effective strategy to address these challenges and provide genetic resources for breeding nitrogen-efficient varieties. *NLP* transcription factors are plant-specific transcription factors that are crucial for nutrient uptake, plant growth and development, stress response, and nitrogen response [12]. Despite their importance, there are few reports on the identification and functional analysis of *NLP* transcription factor gene families in millet. Therefore, we aimed to excavate the critical genes involved in the nitrogen pathway within *NLP* family members to better understand their molecular mechanism in nitrogen utilization, thereby providing a theoretical basis for crop breeding. In this study, we identified seven *NLP* gene family members in the millet genome, systematically analyzing their gene structural characteristics, evolutionary relationships, and replication events. We used low-nitrogen transcriptome data to assess the expression profile of this gene family under low-nitrogen conditions and further examined the expression patterns at different time points under both normal and low-nitrogen conditions using qRT-PCR. Additionally, we investigated the molecular mechanism of key candidate genes involved in the nitrogen pathway through a dual luciferase complementary assay. Furthermore, we utilized 103 core millet germplasm samples with rich genetic diversity to classify the key candidate genes into haplotypes, understand their evolutionary relationships, and track their selection during domestication and improvement. This approach aims to provide valuable genetic resources and a theoretical basis for variety enhancement.

A total of seven *NLP* gene family members have been identified in millet, while relevant studies have reported varying numbers of *NLP* genes in other species [27,28,29]. For instance, nine *NLPs* were identified in *Arabidopsis*, while six *NLPs* were found in rice and tomato. Additionally, the following numbers of *NLPs* were identified in other species: 37 in wheat, 31 in *Brassica napus*, 11 in Asian cotton, 11 in Raymond’s cotton, 22 in upland cotton, 21 in island cotton, 14 in poplar, and 10 in bamboo [26,27,28,29,30,31,32]. Relatively more *NLPs* were identified in wheat, upland cotton, island cotton, and *Brassica napus*, which may be attributed to chromosome doubling during the polyploidy process in these species, resulting in gene duplication [49,50]. The number of *NLPs* identified in millet is comparable to that in rice and tomato. The *NLP* gene family in millet possesses two typical domains, RWP-RK and PB1. With the exception of the *SiNLP7* gene, the other six genes also contain the GAF domain at the N-terminal, which aligns with previous research findings [30]. These domains are known to play significant roles in specifically binding cis-acting elements and facilitating protein interactions in response to nitrates [22,25,39,51,52]. Changes in domain deletion can affect the gene function of *NLPs*, contributing to their diversity in regulating growth, development, and nitrogen pathways [21]. The subcellular localization prediction outcomes reveal that, with the exception of the SiNLP1 protein, which is localized in chloroplasts, all remaining NLP proteins are localized within the nucleus. As a core transcription factor in the nitrogen pathway, *NLP* has been shown to be phosphorylated by calcium-dependent kinases (CPKs), which control its subcellular localization [33]. Some *NLP* genes are found in the cytoplasm, and when nitrate concentrations change, nucleoplasmic shuttling occurs to perform the related functions [53,54]. A similar phenomenon has been observed in other species, for example, *GrNLP8-1* in cotton *NLP* is localized to chloroplasts [30,32,55].

Using *Arabidopsis NLP* as a reference, we conducted evolutionary tree analysis and divided the *NLP* gene family into three subpopulations, with members of the same species preferentially clustered together (Figure 2). These results indicate that the functions of *NLP* gene families may have differentiated somewhat during species evolution. The *NLP* gene family of millet and *Arabidopsis* were each classified into three subgroups, potentially due to functional differentiation among *NLP* gene family members throughout the evolutionary process. Additionally, the evolution of the same gene family across different species may exhibit certain convergences. This subgroup classification aligns with findings from previous research [30]. Moreover, the *SiNLP* gene family was found to contain ten conserved motifs as well as RWP-RK and PB1 domains, suggesting that *SiNLP* genes are highly conserved throughout evolution. Gene structure analysis revealed that the *SiNLP5* gene has the highest number of exons (10) compared to other members, indicating that the function of this gene may have differentiated to some extent during evolution [29]. Cis-acting element analysis indicated a significant presence of regulatory elements related to hormones, stress, light signals, and growth and development in the promoter region. This suggests that the *SiNLP* gene family may be induced by hormone, light, and stress signals during the growth and development of millet, participating in various pathways related to millet’s stress response and overall growth and development. These factors likely play essential roles in regulating plant growth, development, and abiotic stress processes [12,35,36] (Figure 4). Gene replication events promote the expansion of gene families and species evolution [56]. We identified a pair of fragment-replicating colinear genes, *SiNLP3* and *SiNLP4*, located on millet chromosomes three and five, respectively (Figure 5). Collinearity analysis between millet and other species revealed seventeen collinear gene pairs in millet and wheat, and six collinear gene pairs in *Arabidopsis*, rice, and maize. It is possible that the hexaploid nature of wheat resulted in chromosome doubling during species evolution, leading to a rapid expansion of this gene family (Figure 6).

The expression levels of *SiNLP* gene family members vary across different tissues, indicating that the *SiNLP* gene family is involved in various stages of plant growth and development, with spatiotemporal specificity in expression. According to low-nitrogen transcriptome data, five *SiNLP* genes were found to be up-regulated, while two genes were down-regulated, possibly reflecting the different functions of *SiNLP* genes in nitrogen pathways. Notably, the expression of *SiNLP2* was significantly up-regulated following low-nitrogen treatment, with a *p*-value of 1.37 × 10^−36^, suggesting that this gene plays a crucial role in nitrogen pathways. To further investigate the expression of *SiNLP* candidate genes in roots at different time points under normal and low-nitrogen treatment conditions, we conducted qPCR experiments (Figure 7). The results showed that both *SiNLP2* and *SiNLP4* were up-regulated in response to low nitrogen, with varying expression levels at different time points, likely due to the spatiotemporal specificity of their expression. Under low-nitrogen conditions, *SiNLP2* exhibited the most significant changes in expression compared to other genes, reinforcing its critical role in nitrogen pathways. To explore the molecular mechanism of the key candidate gene *SiNLP2* in nitrogen pathways, we predicted the promoter regions of several star genes within the nitrogen pathway [12]. We found that the promoter regions of *SiNAR2.1A*, *SiNAR2.1B*, *SiNRT1.1,* and *SiNR2* all contained binding motifs for *NLP* transcription factors. Additionally, *SiNLP2* can activate the expression of *SiNAR2.1A*, *SiNAR2.1B*, *SiNRT1.1,* and *SiNR2* (Figure 8). These results suggest that *NLP* transcription factors may participate in nitrogen pathways by regulating the expression of related genes. Previous studies have indicated that *NLPs* can regulate the transcription of nitrate-response genes, such as nitrate reductase, *NRT2.1*, and *NRT2.2*, thereby influencing plant nitrogen response [18,19]. Efficient nitrogen absorption, utilization, and assimilation ultimately affect crop yield. To understand the selection of *SiNLP2*, a key candidate gene for low-nitrogen response, during the domestication and improvement of millet, and to identify beneficial haplotypes to provide genetic resources and theoretical support for subsequent variety improvement and molecular breeding, we utilized 103 core germplasm samples with rich genetic diversity. Through multiple-sequence alignment, we established links between the markers and categorized them into two haplotypes (Figure 9A). Interestingly, Hap2 gradually increased during domestication and improved sharply thereafter (Figure 9B). Therefore, we speculate that Hap2 has been selected to varying degrees during domestication and improvement, with greater selection pressure observed during domestication compared to later improvement. Analysis of yield traits among different haplotypes revealed that the thousand-kernel weight, grain weight of the main stem, and panicle weight of the main stem of Hap2 were significantly greater than those of Hap1 (Figure 9C–E). The results indicated that Hap2 is an elite haplotype that is increasingly being utilized in breeding programs.

In summary, we conducted a comprehensive analysis of the structural characteristics, evolutionary relationships, expression profiles, and molecular mechanisms of the *SiNLP* gene family in millet under low-nitrogen conditions. Additionally, we identified the selective characteristics and superior haplotypes of the key candidate gene *SiNLP2*, providing valuable genetic resources and a theoretical basis for variety improvement and marker-assisted selection breeding.

## 4. Materials and Methods

### 4.1. Identification and Physicochemical Properties of NLP Transcription Factor Family Members in Millet

First, we utilized the TAIR database (https://www.arabidopsis.org/browse/gene_family/NLP, Phoenix Bioinformatics Corporation 39899 Balentine Drive, Suite 200 Newark, CA 94560, USA, accessed on 11 August 2024) to download the protein sequences of the *Arabidopsis NLP* gene family. The millet genome sequence, amino acid sequence, and annotation documents were obtained from the Phytozome website (https://phytozome-next.jgi.doe.gov/, U.S. Department of Energy, Joint Genome Institute, Walnut Creek, CA 94598, USA, accessed on 11 August 2024). We selected nine NLP protein sequences from *Arabidopsis* as query sequences and performed a BLAST comparison across the entire cereal genome using TBtools-v2.119 (https://github.com/CJ-Chen/TBtools, South China Agricultural University, Guangzhou City, Guangdong Province, China, accessed on 12 August 2024), with a threshold set to an e-value of less than 1 × 10^−5^. Additionally, we downloaded the RWP-RK (PF02042) and PB1 (PF00564) HMM files for the *NLP* gene family from the Pfam database (http://pfam.xfam.org/, Hinxton, UK, accessed on 14 August 2024). Using the Simple HMM Search tool in TBtools-v2.119, we conducted an HMM search based on the hidden Markov model. We then combined the results from both BLAST and HMM analyses, removing duplicate entries to eliminate redundancy. Furthermore, we validated the identified members of the *NLP* gene family in foxtail millet using SMART-v9.0 website (http://smart.embl-heidelberg.de/, European Molecular Biology Laboratory, Heidelberg, Baden-Württemberg, Germany, accessed on 20 August 2024). The protein parameter calculation tool in TBtools-v2.119 was employed to analyze the number of amino acids, molecular weight (MW), and isoelectric point (pI) of *SiNLP* family members. Finally, we used the Plant-mPLoc website (http://www.csbio.sjtu.edu.cn/bioinf/plant-multi/, Shanghai, China, accessed on 23 August 2024) to predict the subcellular localization of the proteins [57].

### 4.2. Phylogenetic Analysis of the SiNLP Gene Family

Using the *NLP* gene family members identified above, we constructed an evolutionary tree that includes members of the Arabidopsis *NLP* gene family. We began by conducting multiple-sequence alignment of the *NLP* gene family members using MEGA-X-10.1.8 software [58]. Next, we applied the Neighbor-Joining method for the analysis of the evolutionary tree, setting the bootstrap value to 1000. Finally, we enhanced the visualization of the evolutionary tree using ITOL-v7 (https://itol.embl.de/, Heidelberg, Germany, accessed on 24 August 2024).

### 4.3. Analysis of Conserved Motifs, Domains, Cis-Acting Regulatory Elements, and Gene Structure of the SiNLP Gene Family

We used MEME suite 5.5.7 software to analyze motifs (https://meme-suite.org/meme/tools/meme, San Diego, CA, USA, accessed on 24 August 2024), set the motif number to 10, and downloaded the MAST XML output files from the analysis results. The Batch CD Search tool on NCBI was utilized to analyze the domain. We extracted 2000 bp promoter sequences located upstream of the ATG start codons for members of the *SiNLP* gene family from millet genome data. Next, we employed the PlantCARE online tool (https://bioinformatics.psb.ugent.be/webtools/plantcare/html/, Gent, Belgium, accessed on 25 August 2024) to predict the effects of cis-elements on the gene family members. Finally, we used the Gene Structure View (Advanced) tool in TBtools-v2.119 software to visualize the results obtained.

### 4.4. Chromosome Localization and Collinearity Analysis of SiNLP Gene Family Members

The genome sequences, amino acid sequences, and annotation files for *Arabidopsis*, rice (*Oryza sativa*), wheat (*Triticum aestivum*), and maize (*Zea mays*) were obtained from the Phytozome website (https://phytozome-next.jgi.doe.gov/, U.S. Department of Energy, Joint Genome Institute, Walnut Creek, CA 94598, USA, accessed on 27 August 2024). The Gene Location Visualize tool in TBtools-v2.119 was used to visualize the chromosomal localization of the gene family members. Tandem and fragment duplication events among the *SiNLP* gene family members were analyzed using the MCScanX software within TBtools-v2.119. Subsequently, we examined the homology between *SiNLP* gene family members in millet and *NLPs* in *Arabidopsis*, rice, wheat, and maize, utilizing TBtools-v2.119 to display the homology results.

### 4.5. Analysis of Expression Profiles of SiNLP Gene Family Members in Different Tissues and Under Low-Nitrogen Stress

The expression data for the *SiNLP* gene family in different tissues were obtained from the Setaria-db database. The transcriptome data for the millet variety Zheng204 under low-nitrogen stress were sourced from previously published studies related to this experiment. We extracted the expression results of the *SiNLP* gene family members from the transcriptome sequencing data. Finally, we used the HeatMap tool in TBtools-v2.119 to create heat maps.

### 4.6. RNA Extraction and RT-qPCR Analysis of Candidate Genes Under Low-Nitrogen Stress

To determine the expression patterns of candidate genes in the root system at different time points under normal and low-nitrogen conditions, we transplanted germinated Yugu1 seedlings into a 96-well black light-avoiding hydroponic box. The plants were cultured under conditions of 14 h of light and 10 h of darkness, with temperatures of 24 °C during the day and 21 °C at night, and 60% humidity. After three days of growth, we treated the plants with an improved Hoagland solution for normal (CK: 1 mM Ca(NO3)_2_) and low-nitrogen (LN: 0.1 mM Ca(NO3)_2_) conditions. Roots were sampled at 0, 1, 3, 6, and 12 h under both normal and low-nitrogen conditions. Total RNA was extracted from these millet samples using a rapid plant total RNA extraction kit (Zhuangmeng Biotechnology, Beijing, China). The quality and concentration of the RNA were assessed using a NanoDrop 2000 spectrophotometer, and RNA integrity was verified through agarose gel electrophoresis. cDNA was synthesized using a one-step kit (TransScript^®^ One-Step gDNA Removal and cDNA Synthesis SuperMix, TransGen Biotech Co., Ltd., Beijing, China). For qPCR analysis, we utilized a real-time fluorescence quantitative kit (TransStart Top Green qPCR SuperMix (+ Dye II), TransGen Biotech Co., Ltd., Beijing, China), with millet SiActin serving as the internal reference.

### 4.7. DLR Assay

The cis-acting elements in the promoter regions of the *SiNAR2.1A*, *SiNAR2.1B*, *SiNRT1.1*, and *SiNR2* genes were predicted using PlantPAN 4.0 (https://plantpan.itps.ncku.edu.tw/plantpan4/index.html, accessed on 8 September 2024). To construct reporters, we amplified the promoter sequences 2 kb upstream of the ATG of the *SiNAR2.1A*, *SiNAR2.1B*, *SiNRT1.1*, and *SiNR2* genes, respectively, and cloned the promoters into the transient expression vector pGreenII 0800-LUC. Subsequently, the full-length CDS sequence of the *SiNLP2* gene was amplified and cloned into pGreenII 62-SK to generate effectors [59]. In subsequent experiments, the constructed vectors were transferred into foxtail millet protoplasts, and after incubation for 16–18 h, the LUC and REN activities were detected using a Dual-Luciferase Reporter Assay System kit (Promega Corporation, Madison, WI, USA). The Renilla Luciferase (REN) gene was used as an internal control.

### 4.8. Haplotype Analysis of Candidate Genes

To understand the relationship between candidate genes involved in nitrogen use and agronomic traits such as yield, as well as their selection during domestication and improvement, we conducted a haplotype analysis of *SiNLP2*, an essential candidate gene in the *SiNLP* gene family. We utilized 103 core germplasm samples with rich genetic diversity to build a local BLAST database using TBtools-v2.119, from which we extracted the CDS sequences of the candidate genes [60]. Multiple-sequence comparisons were performed using MEGA-X-10.1.8 software, and a heat map was generated using the Heatmap package in R-4.4.1. We performed significance analysis between Hap1 and Hap2 using two-tailed student’s *t*-tests.

## Figures and Tables

**Table 1:** Information on *SiNLP* gene family members in millet.

Name	Gene ID	Protein Length (aa)	Molecular Weight (Da)	Theoretical pI	Instability Index	Aliphatic Index	Grand Average of Hydropathicity	Subcellular localization
*SiNLP1*	Seita.1G094300.1	749	79,534.4	5.38	42.28	78.34	−0.262	Chloroplast
*SiNLP2*	Seita.2G298700.1	886	96,994.84	6.29	47.06	73.97	−0.413	Nucleus
*SiNLP3*	Seita.3G084600.1	894	98,567.06	6	44.32	84.36	−0.264	Nucleus
*SiNLP4*	Seita.5G004100.1	935	102,492.49	5.75	49.19	78.3	−0.36	Nucleus
*SiNLP5*	Seita.6G248300.1	1069	117,460.25	5.67	51.49	74.9	−0.424	Nucleus
*SiNLP6*	Seita.8G074000.1	881	96,301.53	5.83	47.53	79.68	−0.329	Nucleus
*SiNLP7*	Seita.9G553000.1	916	102,330.46	5.41	47.11	74.27	−0.433	Nucleus

## Data Availability

The data presented in this study are available.

## Supplementary Materials

The following supporting information can be downloaded at: https://www.mdpi.com/article/10.3390/ijms252312938/s1.
